# Encephalopathy in children with Dravet syndrome is not a pure consequence of epilepsy

**DOI:** 10.1186/1750-1172-8-176

**Published:** 2013-11-13

**Authors:** Rima Nabbout, Nicole Chemaly, Mathilde Chipaux, Giulia Barcia, Charles Bouis, Celia Dubouch, Dorothee Leunen, Isabelle Jambaqué, Olivier Dulac, Georges Dellatolas, Catherine Chiron

**Affiliations:** 1 Department of Pediatric Neurology, Centre de Reference Epilepsies Rares, Hôpital Necker Enfants Malades APHP, Paris, France; 2 Inserm, U663, Paris F-75015, France; 3 University Paris Descartes, PRES Sorbonne Paris Cité, Paris F-75005, France; 4 CEA, Neurospin, Gif/Yvette 91190, France; 5 Université Paris Descartes, Inserm U669, Maison de Solenn, Paris, France

**Keywords:** Dravet syndrome, Epileptic encephalopathy, Psychomotor delay, Cognitive outcome, *SCN1A* mutation, DQ, IQ

## Abstract

**Background:**

Dravet syndrome (DS) is currently considered as an epileptic encephalopathy, a condition in which epilepsy causes deterioration or developmental delay but preliminary data suggested that cognitive course may worsen independently from epilepsy. Our objective was to prospectively analyze the neuropsychological features in a large cohort of DS patients and its relationships with epilepsy and *SCN1A* mutation.

**Methods:**

81 examinations were performed in 67 patients with typical DS (9m-24y, 15 longitudinally studied) using Brunet-Lezine (developmental/intelligence quotient [DQ/IQ] and DQ sub-scores), Achenbach, Conners, and a semi-quantitative psychomotor score (SQPS). We studied the correlation between DQ/IQ/SQPS and age, epilepsy characteristics, and whether patients presented *SCN1A* mutation.

**Results:**

DQ/IQ significantly decreased with age (r = −.53, p < .001), from normal before 2y (mean 80, range 64–105) to low after 3y (mean 48, range 30–69), with hyperactivity and attention disorders hampering learning abilities especially up to 6y. However, raw (not age-adjusted) DQ sub-scores increased with age during the first decade, showing that there is no regression. We did not find any significant correlation between DQ/IQ at last evaluation and epilepsy data, i.e. first seizure (age, type, duration, fever), seizures during the course (type, fever sensitivity), status epilepticus (age of onset, number, fever), photosensitivity, and treatment, except for myoclonus and focal seizures which were associated with a lower QD/IQ after 3y. *SCN1A* mutated patients (n = 58) seemed to exhibit worse psychomotor course than non-mutated ones (n = 9) (severe SQPS in 26% *vs* 0%), although their epilepsy tended to be less severe (tonic seizures in 12% *vs* 44% [p = 0.04], first status epilepticus before 6 m in 26% *vs* 67% [p = .02], mean number of SE 2.5 *vs* 4.5 [p = .09]). DQ sub-scores were dissociated throughout the whole course: from onset hand-eye coordination was significantly lower than language, posture and sociability (p < .01). Dissociation seemed to be more frequent in mutated than in non-mutated patients (motor SQPS was normal for in 77% *vs* 44% [p = 0.017] whereas language SQPS was normal for 47% *vs* 100%).

**Conclusions:**

Although psychomotor/cognitive delay declines with age, there is no regression. In addition, encephalopathy is not a pure consequence of epilepsy but *SCN1A* mutation seems to play an additional, direct role.

## Background

Dravet syndrome (DS) is a severe epilepsy presenting in the first year of life with clonic seizures triggered by fever, often unilateral and long lasting in a child with previously normal development. Other seizure types may develop between one and four years of age, including myoclonic jerks, focal seizures and atypical absences. Electroencephalogram (EEG) is usually normal at onset, and then shows slowing of basal activity with asymmetrical spikes or polyspikes-waves [1,2]. In addition to pharmacoresistant seizures and frequent episodes of status epilepticus (SE) often triggered by fever, severity of the condition results from the frequent occurrence of progressive worsening of psychomotor delay and sudden death. Mutations in *SCN1A* have been identified in 75% of DS patients [3], and mutations in *PCDH19* were found in 10% of the *SCN1A*-negative population [4].

Developmental delay in DS is usually considered as a result of severe epilepsy, and the term “epileptic encephalopathy” therefore often applied [5-7]. Occurrence of SE and early spikes on EEG proved to be predictors of a worse developmental outcome in a large DS cohort of *SCN1A* mutated patients [8]. However preliminary data question this relationship between cognitive outcome and the course of epilepsy. Two patients with *SCN1A* truncation and severe cognitive decline presented with divergent epilepsy severity [9]. Cognitive scores were not related to the frequency of convulsive seizures in a series including 16% of *SCN1A*-negative patients [10]. DS patients with *PCDH19* mutations seem to develop better than those with *SCN1A* mutations despite the occurrence of explosive convulsive clusters [4]. The first attempt to detect any difference in cognitive outcome according to the presence or not of *SCN1A* mutation was unsuccessful [11].

Whether Nav.1 dysfunction plays a role in the cognitive decline independently from the epilepsy has not been proved so far. While epilepsy features in children affected by DS have been described in detail for a long time [1,12,13], neurodevelopmental features were only recently addressed and available data mostly consist of small and/or retrospective series [7-11,14,15], sometimes without formal neuropsychological testing [8].

The aim of this study was to analyze the neuropsychological and behavioral features prospectively in a large cohort of patients with DS and its relation with *SCN1A* mutation and the characteristics of epilepsy.

## Methods

All children and adolescents followed at the Neuropediatric Department of Necker Hospital, with clinical and EEG features of typical DS, based on the ILAE classification [16], were prospectively enrolled in this study from January 2006. Inclusion criteria were the following: i) normal infant with normal EEG and without preexisting cerebral lesion, developing normally until the first seizure occurring before one year of age, ii) refractory clonic or tonic-clonic seizures affecting one or both sides simultaneously or alternatively, with eventually additional seizure types during follow-up, iii) exclusion of any other identified epilepsy syndrome. All *SCN1A* negative patients were tested for *PCDH19* and those with *PCDH19* mutation were excluded.

Clinical data were assessed prospectively from 2006 to 2010 by the same three pediatric neurologists (RN, OD, CC). Those obtained before 2006 were retrieved from the medical files. Demographic data included familial (history of febrile seizures/epilepsy) and personal (pre- and peri-natal) antecedents. Epilepsy data (used as variables) included first seizure (age, febrile/afebrile, status epilepticus [SE]/non SE [the term SE designated seizures lasting more that 30 minutes], unilateral/generalized), seizures during course (presence or absence of tonic-clonic, clonic, tonic, absences, myoclonic or focal seizures and sensitivity to fever or not), SE (presence or absence of SE, and if any: age at first SE, number of SE, sensitivity to fever or not), photosensitivity (yes/no on EEG), and treatment (therapy used, age at stiripentol introduction if any, use or not of any antiepileptic drugs considered inappropriate for DS and duration if any). Neurological data included gait and neurological examination.

Mutation analysis of the *SCN1A* was performed by direct sequencing followed, in negative patients, by multiplex ligation probe amplification (MLPA) as reported previously [4]. Patients were considered mutated when they presented any abnormality on *SCN1A* gene (mutation, rearrangement, deletion) and “non-mutated” when they had no abnormality detectable on *SCN1A*.

Neuropsychological evaluations were prospectively performed by the same neuropsychologist from 2006. Raw data of evaluations performed before 2006 were reanalyzed and used for this study. Neurodevelopmental features and adaptation were assessed according to age and level of collaboration using Wechsler Scales (WPPSI, WISC-IV) (IQ, intellectual quotient) [17,18], Brunet-Lezine Developmental Scale (DQ, developmental quotient) with sub-scores to be completed up to 6 years (the superior age at which the Brunet-Lezine scale is applicable) [19]. Behavior troubles were screened by clinical observation, parent’s interview, Achenbach Child Behavior Checklist and Conners Rating Scale [20,21]. In addition, psychomotor development was assessed by the pediatric neurologist with the neuropsychologist during free play using a semi-quantitative psychomotor scale (SQPS) (no/moderate/severe delay) for global, motor and language development.

For the present analysis, we used all the DQ/IQ and DQ sub-scores obtained during the whole follow-up, whereas Achenbach, Conners and SQPS were collected from the last evaluation.

We studied the relationship between psychomotor/cognitive/behavioral profile, age, epilepsy, and genetic background. First we studied the relationship between DQ/IQ scores, DQ sub-scores, Achenbach, and Conners, on one hand, and age on the other hand. A special analysis of the changes with age in DQ/IQ and DQ sub-scores was performed in the sub-population of patients longitudinally assessed. Secondly we studied the relationships between DQ/IQ and epilepsy variables. Thirdly we studied the relationships between the presence or absence of *SCN1A* abnormality on one hand, and neuro-developmental scores at last evaluation (DQ/IQ, DQ sub-scores, and semi-quantitative psychomotor assessment) and epilepsy variables on the other hand. Statistical analyses were performed using bivariate and univariate tests: correlation for continuous variables, chi-square and if necessary Fisher exact for categorical variables, and t-test and if necessary signed rank test to compare means.

### Patients

The clinical, cognitive and genetic data are summarized in (Table 1).

**Table 1:** Clinical, cognitive and genetic data

**Clinical and cognitive characteristics**	**SCN1A+**	**SCN1A-**	**Total**
**N**	58	9	67
**Sex**: boys N (%)	33 (56.9)	6 (66.7)	39 (58.2)
**Age in years**: mean (SD)			
at last follow-up	9.1 (5.3)	8.7 (4.6)	9.0 (5.2)
at first examination	4.0 (5.4)	4.0 (2.7)	4.0 (5.2)
**Follow-up duration**	5.1 (4.2)	4.7 (5.6)	5.0 (4.4)
**Family history for seizures**: N (%)	21 (36.2)	4 (44.4)	25 (37.3)
Dravet Syndrome	3 (5.2)	0	3 (4.5)
Febrile seizures	14 (24.1)	3 (33.3)	17 (25.4)
Febrile seizures plus	2 (3.4)	0	2 (3.0)
Epilepsy	8 (13.8)	1 (11.1)	9 (13.4)
**Age at first seizure**: N (%)			
0-3 months	6 (10.3)	1 (11.1)	7 (10.4)
3-6 months	29 (50.0)	5 (55.6)	34 (50.7)
6-9 months	18 (31.0)	1 (11.1)	19 (28.4)
> 9 months	5 (8.3)	2 (22.2)	7 (10.5)
**Type of seizures**: N (%)			
Tonic-clonic	51 (87.9)	8 (88.9)	59 (88.1)
Absences	32 (55.2)	5 (55.6)	37 (55.2)
Myoclonic	29 (50.0)	6 (66.7)	35 (52.2)
Tonic	7 (12.1)	4 (44.4)	11 (16.4)
Clonic	38 (65.5)	7 (77.8)	45 (67.2)
Partial	23 (39.7)	3 (33.3)	26 (38.8)
**Sensitivity to fever**	51 (87.9)	7 (77.8)	58 (86.6)
**Photosensitivity**	11 (19.0)	4 (44.4)	15 (22.4)
**Status Epilepticus** (SE)			
Present: N (%)	46 (79.3)	6 (66.7)	52 (77.6)
**Age at first SE**: N (%)			
0-3 months	0	1 (16.7)	1 (1.9)
3-6 months	12 (26.1)	3 (50.0)	15 (28.8)
6-9 months	14 (30.4)	0	14 (26.9)
9-12 months	7 (15.2)	1 (16.7)	8 (15.4)
>12 months	13 (28.3)	1 (16.7)	14 (26.9)
**Neurological features**: N (%)			
Ataxia/Gait disorders*	45 (77.5)	4 (44)	49 (73)
Pyramidal signs	4 (6.9)	1 (11.1)	5 (7.5)
**Last DQ/IQ**			
Mean (SD)	53.4 (20.9)	42.9 (11.3)	51.9 (20.2)
Range	30-105	30-65	30-105
Age at last DQ/IQ (years)			
Mean (SD)	6.0 (4.6)	8.7 (3.7)	6.4 (4.5)
Range	1.1-23.9	4.6-16.1	1.1-23.9
**Semi-quantitative psychomotor score**			
(SQPS)			
Mild	33 (57.9)	5 (55.6)	38 (57.6)
Moderate	9 (15.8)	4 (44.4)	13 (19.7)
Severe	15 (26.3)	0	15 (22.7)

N = number, SD = standard deviation, DQ/IQ = developmental/intellectual quotient. *p = 0.017.

#### Demographics

The cohort consisted of 67 patients (39 males and 28 females) with a median age of nine years at the time of last follow-up (range: 1.8 – 24 years). The mean follow-up was five years.

A positive family history for seizures was reported in 25 patients: DS in three (two sibs and the brother of an affected sister not included), febrile seizures in 19, other epilepsies in nine. Seizures phenotype, cognitive and behavioural profile were not similar in both DS sibs. Personal antecedents included intrauterine growth delay in three patients (4%), neonatal respiratory distress in three (4%), and prematurity in two (3%).

#### Clinical data

All patients presented the first seizure between one and 12 months of age, mostly between three and six months. The first seizure was unilateral clonic in 25 patients (37%). It was long-lasting, evolving to SE in 23 (34%).

During follow-up, seizures were polymorphous in all patients, mainly nocturnal in 12 of them, and 15 patients presented with clinical photosensitivity. Seizures were mostly tonic-clonic or clonic, atypical absences and myoclonic seizures were present in about half the cases, and 16% of patients experienced tonic seizures. SE was reported in 78% of patients, occurring up to the age of 8 years, 79% of SE occurred during the first year of life. SE and shorter seizures were triggered by fever in respectively 70% and 87% of the children.

At last examination, gait disturbance affected 49 patients (73%) and pyramidal signs five (7%). Two children died, respectively at the ages of 3.5 and 6.5 years: the first in a context of SUDEP during sleep with no witnessed seizure, and the second in the course of a brainstem tumor that was refractory to chemotherapy and offered no surgical possibility.

#### Treatment

Fifty six patients (84%) received the valproate, stiripentol and clobazam (VPA-STP-CLB) tri-therapy. Thirty patients received additional antiepileptic drugs, mainly topiramate or levetiracetam (45%), and the ketogenic diet (23%). A few patients received zonisamide (8%) or clonazepam (6%). Seven patients (10%) did not receive stiripentol but the combination of valproate, clobazam and topiramate.

Stiripentol was introduced at a median age of 28 months (range: 0.5-12 years), in adjunction to valproate and clobazam in 43 patients, or in adjunction to valproate and concomitantly to clobazam in 11 patients. In 2 patients, the 3 drugs were introduced together, after the first SE.

Seventeen patients (25%) received during the first three years of life drugs that could be considered inappropriate for this condition: carbamazepine in 11 patients, lamotrigine in 8 and vigabatrin in 4. All patients treated with these drugs experienced worsening of seizure frequency and for all it was withdrawn within less than three months.

#### Genetics

Mutations and rearrangements in *SCN1A* were found in 58 patients (86%). Mutations were *de novo* in 92% of cases, including missense mutations in 18 (26%), and frameshift or non-sense mutations in 40 (60%). MLPA identified no deletions in patients with no mutations.

### Consent

Neuropsychological evaluation was part of the usual clinical work-up of Dravet patients in our practice. Informed consent was obtained from the patient’s parents for the publication of this report.

## Results

We performed a total of 81 neuropsychological evaluations with Wechsler or Brunet-Lezine Scales in this series (67 patients). In 10 patients (15%) such evaluation was not possible because of opposing (7 cases) or autistic (3 cases) behavior, and we could score 85% of our series. We also obtained responses for Conners scale in 58 patients and for Achenbach in 59.

### Cognitive development according to age

#### Overall series

**Figure 1:**
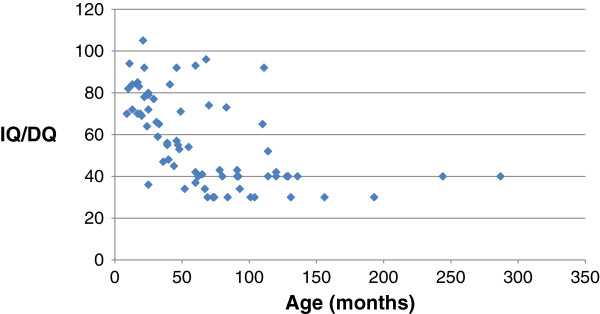
Developmental/intelligence quotient in the whole population.

Among all evaluations, a significant decrease of DQ or IQ with increasing age was observed (r = −.53, p < .001; Spearman’s Rho = −.66, p < .001) (Figure 1). Up to the age of three years mean DQ/IQ remained generally above 70 (before two years of age mean = 79.5, SD = 12.0, range 64–105 and before three years mean = 73.7, SD = 15.0, range 36–105). There was a strong decrease after the age of three (mean = 48.0, SD = 18.9, range 30–69). Only four patients remained with an IQ above 60 after the age of five; all four had *SCN1A* mutation that was inherited for only the fourth patient.

One had an IQ of 93 at 5.4 years and at 73 at 6.9 years. He presented three episodes of SE during the first two years and received the tri-therapy (VPA-STP-CLB) since the age 18 months. He was almost free of seizures till the age of five and presented frequent nocturnal tonic-clonic seizures since this age. He attended normal school program with a personalized assistant teacher. The second patient had an IQ of 74 at 5.8 and 65 at nine years. He required personalized support in school and was to be addressed to a special educational program. He had frequent episodes of SE and frequent polymorphous seizures on VPA-STP-CLB. The third patient had an IQ of 96 at the last evaluation at 5.6 years, and was still in the main school stream. He had rare episodes of SE and became almost seizure free on topiramate (he required no stiripentol). The last patient had an IQ of 92 at nine years of age, and attended normal school. He had frequent febrile seizures, two episodes of SE and frequent atypical absences with generalized tonic-clonic seizures. Seizures were controlled with valproate and clobazam. He presented behavior troubles.

All patients other than these four were following a special educational program out of the normal school.

The Brunet-Lezine (children less than 6 years) sub-scores disclosed heterogeneity between the four tested fields: hand-eye coordination (oculo-motor), language, posture and sociability. Hand-eye coordination DQ sub-score (mean = 62.0, SD = 16.8) was significantly lower (p < .01, signed rank test) than respectively language (mean = 72.3, SD = 17.6), socialization (73.4, SD = 15.8) and posture (mean = 76.5, SD = 16.9) DQ sub-scores. Difference between language, posture and sociability skills was not significant. All patients had acquired language at last evaluation and those over six years were able to build sentences. All could walk with a certain extent of gait disturbance except for one who required a wheel chair.

**Figure 2:**
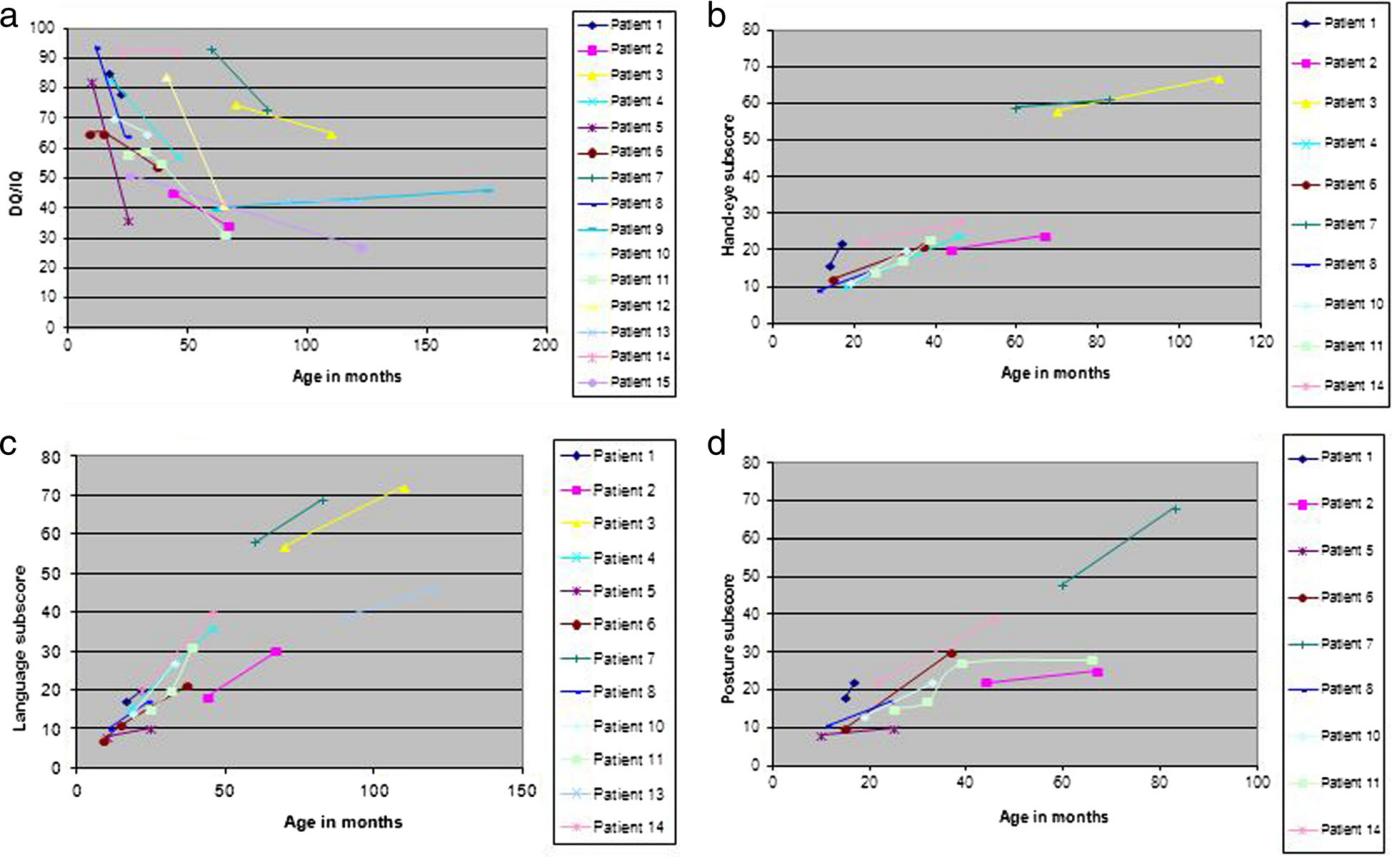
Course of the developmental/intelligence quotient and sub-scores.

The Achenbach scale showed abnormal sub-scores identifying social problems and attention deficit. Although the mean scores remained under 70, they were in the gray area for both fields (T-score between 60 and 70). The Conners scale showed abnormal learning abilities (>70) and a hyperactivity score in the gray area (Figure 2). These findings were concordant with the clinical evaluation of these patients that pointed to hyperactivity and attention deficit, namely between two and five years of age, when the Conners test exhibited the highest pathological hyperactivity score (>70). Twenty percent of the patients mainly within this age range required methylphenidate, with clinical and scores’ improvement. Although some autistic features were reported by parents and educators and on clinical observation, mainly consisting of repetitive body movements, ritualistic behavior and poor eye contact, these symptoms were often isolated and patients did not fulfill the clinical criteria for autism except in 3 cases.

#### Longitudinal series

Fifteen patients had two neuropsychological evaluations. The first evaluation was performed at a mean age of 34 months (SD = 22, range 9–91), and the second at a mean age of 66 months (SD = 43, range 15–175). DQ/IQ at first evaluation was significantly higher than at second evaluation (mean of the difference = 15.1, SD = 19.8, p < .01 signed rank test). Twelve out of 15 patients exhibited a decline DQ/IQ pattern similar to the overall series (Figure 2a). Only two of the 15 remained with DQ/IQ over 60 after 5 years of age. Although IQ/DQ score continued dropping in all 15 patients, they continued acquiring new abilities as disclosed by persistent although slow increase in the absolute values of DQ sub-scores (Figure 2b,c,d). This slow progress was also clinically obvious.

#### Cognitive development according to epilepsy

For overall evaluations, statistical analysis failed to detect any significant correlation between global DQ/IQ and epilepsy variables, i.e. first seizure (age, type, duration, fever), seizures during the course (type, fever sensitivity), SE (age of onset, number, fever), photosensitivity, and treatment (age at stiripentol introduction, administration of an inappropriate medication for less than three months).

However, for patients over the age of three years (i.e. when the cognitive delay is almost constant), IQ/DQ was significantly lower for patients exhibiting myoclonic (p = .03) or focal seizures (p = .03). This remained significant when only the mutated children were considered (notice that none of the nine non-mutated patients were evaluated before three years of age in the present series).

#### Cognitive development and epilepsy according to genetics

There was no difference for demographic data, the age at first and last observation and for the duration of follow-up between patients with and without *SCN1A* mutation. However, the occurrence of another DS case in the family was only reported in the *SCN1A*-mutated group and the patients with intrauterine growth retardation were all three mutated.

Although there was no significant difference for last DQ/IQ according to whether there was or not *SCN1A* mutation, the mutated and non-mutated groups exhibited different psychomotor outcome according to their pediatric neurologist (Table 1). The semi-quantitative evaluation (SQPS) showed that no patient had severe delay in the non-mutated group whereas all patients labeled severe delay (26%) were in the mutated group. All the patients classified with severe delay using this scale had DQ/IQ < 50.

Similarly, gait and language skills tended to be more favorable in the non-mutated group. Gait was considered normal by the clinician in 44% of the non-mutated patients but abnormal in 77.5% of *SCN1A* + group (p = 0.017). Language was normally structured in all *SCN1A*- patients but only in 47% (27/58) of *SCN1A* + patients. However, given the small size of the non-mutated group, these differences did not reach statistical significance.

Regarding epilepsy, there was no difference between both groups for first seizure (age, type, fever), photosensitivity and treatment, but tonic seizures were more frequent in the non-mutated than in the mutated patients (Fisher exact: p = .04). In addition, age at first SE tended to be lower in the non mutated group: SE occurred before 6 months in 67% of the non mutated patients *vs* 26% of the mutated ones (Fisher exact: p = .02). SE also tended to be more frequent in the none mutated than the mutated group (mean number 4.4 *vs* 2.5, p = .09).

## Discussion

Based on a large and homogeneous population of children with typical DS mostly prospectively and partly longitudinally assessed for cognitive development, this study shows that although retardation worsens with age, there is no loss of abilities and patients make slow but regular progress along the first decade. As suspected, cognitive outcome is related to epilepsy course and seizure characteristics since myoclonia and focal seizures are associated with a lower QD/IQ level after 3 years of age. However, epilepsy does not account for the whole cognitive picture since mutated patients tend to exhibit worse psychomotor course than non-mutated ones although epilepsy tends to be less severe. *SCN1A* mutation is therefore a key factor of cognitive delay, in addition to epilepsy. We also observe a dissociated cognitive profile, particularly for patients with *SCN1A* mutation: speech develops better than visuo-motor function, from the first years of life and before cognitive slowing that occurs mainly after age three.

### Methodological issues

Although many scales have been developed and validated to investigate cognitive functions, none is appropriate for patients who cannot follow regular schooling. Scales for psychomotor development (Brunet-Lezine and Denver) are most sensitive to age and lack sensitivity in the context of slow but protracted acquisitions [19]. Scales for intelligence (WISC and WPPSI) lose sensitivity under IQ values of 50, and subtests for DQ/IQ require minimal scores of 60 to be performed [17,18]. Other scales developed for severely delayed children do not address cognition. The Vineland scale evaluates adaptation to the surrounding and is very sensitive to parents’ level of education, which precludes reliable comparison between patients [22]. The same holds for quality of life scales [21]. In order to overcome this challenge, we applied several scales to each child and focused on the youngest children since they had made enough acquisitions to be comparable to their age mates.

### The lack of psychomotor deterioration

DS patients usually experience a slowing of cognitive achievements that becomes evident after the first year of life, and they reach the low DQ/IQ level by five years of age [7,8,10]. Whether the patients then continue deteriorating or stabilize at this low level has been debated. Data drawn from a first prospective study in 12 subjects indicated stabilization in all of them, including the six with typical DS [14]. Our findings show that although the condition worsens progressively compared to healthy children there is no loss of abilities since all patients acquired new skills during follow-up. Obviously, this does not apply to the rare patients who had developed major anoxic-ischemic sequelae after severe and complicated status epilepticus with atrophy of supratentorial structures [23] or lesions in the subcortical structures [24] and who were therefore not included in the present study.

### Epilepsy cannot completely explain the cognitive course

DS is often referred to as an epileptic encephalopathy [5-8]. Indeed, there is progressive worsening of the cognitive defect along the occurrence of pharmacoresistant epileptic seizures and in two series cognitive impairment was linked to the frequency of convulsive seizures [7] and the presence of SE or EEG spikes [8]. However, discordant results have emerged from one case report and a retrospective series [9,10]. Our series also found no correlation between the severity of cognitive delay and that of seizure activity. As in Ragona’s study [10], cognitive scores were only related to non-convulsive seizures for the sub-population of patients over three years of age, thus when patients were the most delayed. Minor seizures consisted of myoclonus and “absences” in Ragona’s series [11], but of myoclonus and complex partial seizures in ours. However, the distinction between “absences” and “complex partial seizures” may be debatable, given the difficulty performing ictal EEG records in such poorly cooperating children and adolescents. Moreover, in our series, patients without *SCN1A* mutation tended to be more preserved than the mutated ones for both motor and speech skills, although their epilepsy characteristics were those that are usually associated with poor cognitive outcome: tonic seizures, earlier onset of SE and higher number of SE, thus hallmarks of severe epileptic encephalopathy [25].

DS does therefore not correspond to the usual definition of epileptic encephalopathy, i.e. worsening of function as a consequence of epileptic activity itself. It is possible that *SCN1A* alteration *per se* plays a role in psychomotor delay, affecting structures/pathways not involved in epilepsy. Although we cannot exclude other genetic factors, this finding should encourage the search of alternative explanations for the motor and cognitive delay, the identification of which could offer more appropriate targets for future treatment.

### SCN1A mutation likely plays a central role on other pathways than those devoted to epilepsy

The dissociated cognitive profile is a major characteristic of DS patients, consisting in a worse impact on visuo-spatial than speech abilities, found in our series as in two previous reports [7,10]. Main troubles include visual motor integration and visual perception, and are associated with impairment of attention. Furthermore, in our series as in Chieffo et al’s patients, visuomotor disorders were detected from the first year of life, several months before the cognitive decline could be identified [26]. Since this trouble was not related to age, the hypothesis of a maturational delay - maturation of the right hemisphere is known to precede that of the left hemisphere in right-handed healthy infants [27] – is excluded. Hypothesis of cerebellar dysfunction has been proposed [26].

Another marker of possible cerebellar dysfunction is ataxia, a component of the clinical pattern from the first steps of patients with DS [1,2], even before any episode of SE has occurred, and before the administration of any medication that could account for ataxia as a side effect, such as benzodiazepines. Myoclonus also is often associated with cerebellar dysfunction, in animal models as in human disease, in progressive and non-progressive conditions [28-31].

Considering that Nav 1.1, the protein determined by the *SCN1A* gene, is expressed in the initial segment of inhibitory neurons of the cerebellum in addition to the cerebral cortex, in transgenic mice [32], cerebellar dysfunction is likely a constitutive component of DS and could contribute to the occurrence of altogether the myoclonus, the gait disorders and the cognitive delay.

This study shows that although the psychomotor/cognitive delay worsens with age, there is no regression in DS patients. Their encephalopathy is not a pure consequence of epilepsy, but *SCN1A* mutation seems to play an additional direct role. Altogether, these results plead for DS encephalopathy being genetic as well as epileptic in origin.

## Abbreviations

DS: Dravet syndrome; EEG: Electroencephalogram; SE: Status epilepticus; DHPLC: Denaturing high performance liquid chromatography; MLPA: Multiplex ligation probe amplification; WISC: Wechsler intelligence scale for children; WPPSI: Wechsler preschool and primary scale of intelligence; IQ: Intellectual quotient; DQ: Developmental quotient; SQPS: Semi-quantitative psychomotor scale; VPA: Valproate; STP: Stiripentol; CLB: Clobazam; SD: Standard deviation.

## Competing interests

The authors declare that they have no competing interest.

## Authors’ contributions

RN conceived the study design, followed the patients, made the interpretation of the results, and wrote the manuscript. NC followed patients and analyzed the results. MC followed patients and participated in the results analysis. GB participated in the analysis of the results. CB, CD and DL performed the neuropsychological evaluations of the patients. IJ participated to conceive the study and made the supervision of the neuropsychological part of the paper. GD performed statistical analyses. OD followed and referred patients and extensively reviewed the manuscript. CC participated to conceive the study, followed and referred patients, and drafted the manuscript. All authors read and approved the final manuscript.

## Authors’ information

RN (MD, PhD) is the coordinator of the Reference Centre of Necker-Enfants Malades Hospital (Paris, France) and the head of the Group *Phenotype/Genotype* in the Research Unit (U663) *Paediatric Epilepsies and Brain Plasticity*. NC, MC, GB are paediatric neurologists and CB, CD, DL are neuropsychologists at the Reference Centre of Rare Epilepsies (NC and GB also are PhD students). IJ (PhD) is head of the Group *Neuropsychology* in the Research Unit. OD (MD) was head of the Neuropediatric department of Necker Hospital. GD (PhD) is researcher in epidemiology and statistics. CC (MD, PhD) is the head of the Research Unit *Paediatric Epilepsies and Brain Plasticity*.

## References

[B1] Dravet C Bureau M Oguni H Roger J, Bureau M, Dravet C, Genton P, Tassinari CA, Wolff P Severe Myoclonic Epilepsy in Infancy (Dravet Syndrome) Epileptic Syndromes in Infancy, Childhood and Adolescence 2002 London: John Libbey 75 88

[B2] Dravet C Bureau M Oguni H Fukuyama Y Cokar O Severe myoclonic epilepsy in infancy: Dravet syndrome Adv Neurol 2005 95 71 102 15508915

[B3] Claes L Del-Favero J Ceulemans B Lagae L Van Broeckhoven C De Jonghe P De novo mutations in the sodium-channel gene SCN1A cause severe myoclonic epilepsy of infancy Am J Hum Genet 2001 68 1327 1332 10.1086/320609 11359211 PMC1226119

[B4] Depienne C Bouteiller D Keren B Cheuret E Poirier K Trouillard O Benyahia B Quelin C Carpentier W Julia S Afenjar A Gautier A Rivier F Meyer S Berquin P Helias M Py I Rivera S Bahi-Buisson N Gourfinkel-An I Cazeneuve C Ruberg M Brice A Nabbout R Leguern E Sporadic infantile epileptic encephalopathy caused by mutations in PCDH19 resembles Dravet syndrome but mainly affects females PLoS Genet 2009 5 e1000381 10.1371/journal.pgen.1000381 19214208 PMC2633044

[B5] Nabbout R Dulac O Epileptic syndromes in infancy and childhood Curr Opin Neurol 2008 21 2 161 166 10.1097/WCO.0b013e3282f7007e 18317274

[B6] Catarino CB Liu JY Liagkouras I Gibbons VS Labrum RW Ellis R Woodward C Davis MB Smith SJ Cross JH Appleton RE Yendle SC McMahon JM Bellows ST Jacques TS Zuberi SM Koepp MJ Martinian L Scheffer IE Thom M Sisodiya SM Dravet syndrome as epileptic encephalopathy: evidence from long-term course and neuropathology Brain 2011 134 2982 3010 10.1093/brain/awr129 21719429 PMC3187538

[B7] Wolff M Casse-Perrot C Dravet C Severe myoclonic epilepsy of infants (Dravet syndrome): natural history and neuropsychological findings Epilepsia 2006 47 Suppl 2 45 48 17105460 10.1111/j.1528-1167.2006.00688.x

[B8] Brunklaus A Ellis R Reavey E Forbes GH Zuberi SM Prognostic, clinical and demographic features in SCN1A mutation-positive Dravet syndrome Brain 2012 135 2329 2336 10.1093/brain/aws151 22719002

[B9] Riva D Vago C Pantaleoni C Bulgheroni S Mantegazza M Franceschetti S Progressive neurocognitive decline in two children with Dravet syndrome, de novo SCN1A truncations and different epileptic phenotypes Am J Med Genet 2009 149A 2339 2345 10.1002/ajmg.a.33029 19764027

[B10] Ragona F Brazzo D De Giorgi I Morbi M Freri E Teutonico F Gennaro E Zara F Binelli S Veggiotti P Granata T Dravet syndrome: early clinical manifestations and cognitive outcome in 37 Italian patients Brain Dev 2010 32 71 77 10.1016/j.braindev.2009.09.014 19854600

[B11] Ragona F Granata T Dalla Bernardina B Offredi F Darra F Battaglia D Morbi M Brazzo D Cappelletti S Chieffo D De Giorgi I Fontana E Freri E Marini C Toraldo A Specchio N Veggiotti P Vigevano F Guerrini R Guzzetta F Dravet C Cognitive development in Dravet syndrome: a retrospective, multicenter study of 26 patients Epilepsia 2011 52 386 392 21269283 10.1111/j.1528-1167.2010.02925.x

[B12] Caraballo RH Fejerman N Dravet syndrome: a study of 53 patients Epilepsy Res 2006 70 Suppl 1 231 238 10.1016/j.eplepsyres.2005.11.026 16893627

[B13] Marini C Mei D Temudo T Ferrari AR Buti D Dravet C Dias AI Moreira A Calado E Seri S Neville B Narbona J Reid E Michelucci R Sicca F Cross HJ Guerrini R Idiopathic epilepsies with seizures precipitated by fever and SCN1A abnormalities Epilepsia 2007 48 1678 1685 10.1111/j.1528-1167.2007.01122.x 17561957

[B14] Chieffo D Battaglia D Lettori D Del Re M Brogna C Dravet C Mercuri E Guzzetta F Neuropsychological development in children with Dravet syndrome Epilepsy Res 2011 95 1–2 86 93 21474289 10.1016/j.eplepsyres.2011.03.005

[B15] Guzzetta F Cognitive and behavioral characteristics of children with Dravet syndrome: an overview Epilepsia 2011 52 Suppl 2 35 38 21463277 10.1111/j.1528-1167.2011.02999.x

[B16] Berg AT Berkovic SF Brodie MJ Buchhalter J Cross JH van Emde BW Engel J French J Glauser TA Mathern GW Moshé SL Nordli D Plouin P Scheffer IE Revised terminology and concepts for organization of seizures and epilepsies: report of the ILAE Commission on Classification and Terminology, 2005–2009 Epilepsia 2010 51 4 676 685 10.1111/j.1528-1167.2010.02522.x 20196795

[B17] Wechsler D WPPSI-III: Echelle d’Intelligence de Wechsler pour la période Préscolaire et Primaire 2004 Paris: Les Editions du Centre de Psychologie Appliquée

[B18] Wechsler D WISC-IV: Echelle d’Intelligence de Wechsler pour Enfants 2005 Paris: Les Editions du Centre de Psychologie Appliquée

[B19] Joose D Echelle de Développement Psychomoteur de la Première enfance 2001 Brunet-Lézine Révisé. Editions et Applications Psychologiques

[B20] Achenbach T Manual for the Child Behavior Checklist/4–18 and 1991 Profile 1991 Department of Psychiatry: University of Vermont, Burlington, VT, USA

[B21] Conners CK Conners’ Rating Scales 1997 New York: RevisedMulti Health Systems

[B22] Sparrow SS Carrey NJ Wiggins DM Millin RP Hosendocus SN Vineland Adaptive Behavior Scales 1984 Circle Pines, Minn: American Guidance Service

[B23] Chipaux M Villeneuve N Sabouraud P Desguerre I Boddaert N Depienne C Chiron C Dulac O Nabbout R Unusual consequences of status epilepticus in Dravet syndrome Seizure 2010 19 190 194 10.1016/j.seizure.2010.01.007 20172746

[B24] Tsuji M Mazaki E Ogiwara I Wada T Iai M Okumura A Yamashita S Yamakawa K Osaka H Acute encephalopathy in a patient with Dravet syndrome Neuropediatrics 2011 42 78 81 10.1055/s-0031-1279725 21647847

[B25] Parisi P Spalice A Nicita F Papetti L Ursitti F Verrotti A Iannetti P Villa MP “Epileptic encephalopathy” of infancy and childhood: electro-clinical pictures and recent understandings Curr Neuropharmacol 2010 8 409 421 10.2174/157015910793358196 21629447 PMC3080596

[B26] Chieffo D Ricci D Baranello G Martinelli D Veredice C Lettori D Battaglia D Dravet C Mercuri E Guzzetta F Early development in Dravet syndrome; visual function impairment precedes cognitive decline Epilepsy Res 2011 93 73 79 10.1016/j.eplepsyres.2010.10.015 21109403

[B27] Chiron C Jambaque I Nabbout R Lounes R Syrota A Dulac O The right brain hemisphere is dominant in human infants Brain 1997 120 1057 1065 10.1093/brain/120.6.1057 9217688

[B28] Ishino H Higashi S Chuta M Ohta H Juvenile Alzheimer’s disease with myoclonus: amyloid plaques and grumose alteration in the cerebellum Clin Neuropathol 1984 3 193 198 6499295

[B29] Bhatia KP Brown P Gregory R Lennox GG Manji H Thompson PD Ellison DW Marsden CD Progressive myoclonic ataxia associated with coeliac disease. The myoclonus is of cortical origin, but the pathology is in the cerebellum Brain 1995 118 1087 1093 10.1093/brain/118.5.1087 7496772

[B30] Abbott LC Bump M Brandl A De Laune S Investigation of the role of the cerebellum in the myoclonic-like movement disorder exhibited by tottering mice Mov Disord 2000 15 Suppl 1 53 59 10755273 10.1002/mds.870150710

[B31] Koh KN Lim BC Hwang H Park JD Chae JH Kim KJ Hwang YS Kim SK Wang KC Moon HK Cerebellum can be a possible generator of progressive myoclonus J Child Neurol 2010 25 728 731 10.1177/0883073809342273 19773463

[B32] Lorincz A Nusser Z Cell-type-dependent molecular composition of the axon initial segment J Neurosci 2008 28 14329 14340 10.1523/JNEUROSCI.4833-08.2008 19118165 PMC2628579

